# The Effect of Alpha Mangostin on Epithelial-Mesenchymal Transition on Human Hepatocellular Carcinoma HepG2 Cells Surviving Sorafenib via TGF-β/Smad Pathways

**DOI:** 10.34172/apb.2020.078

**Published:** 2020-08-09

**Authors:** Syarinta Adenina, Melva Louisa, Vivian Soetikno, Wawaimuli Arozal, Septelia Inawati Wanandi

**Affiliations:** ^1^Master Program in Biomedical Sciences, Faculty of Medicine, Universitas Indonesia.; ^2^Department of Pharmacology and Therapeutics Faculty of Medicine, Universitas Indonesia.; ^3^Department of Biochemistry and Molecular Biology, Faculty of Medicine, Universitas Indonesia.

**Keywords:** Hepatocellular carcinoma, Alpha mangostin, Sorafenib, TGF-β, Epithelial-mesenchymal transition (EMT)

## Abstract

***Purpose:*** This study was intended to find out the impact of alpha mangostin administration on the epithelial-mesenchymal transition (EMT) markers and TGF-β/Smad pathways in hepatocellular carcinoma Hep-G2 cells surviving sorafenib.

***Methods:*** Hepatocellular carcinoma HepG2 cells were treated with sorafenib 10 μM. Cells surviving sorafenib treatment (HepG2^surv^) were then treated vehicle, sorafenib, alpha mangostin, or combination of sorafenib and alpha mangostin. Afterward, cells were observed for their morphology with an inverted microscope and counted for cell viability. The concentrations of transforming growth factor (TGF)-β1 in a culture medium were examined using ELISA. The mRNA expressions of TGF-β1, TGF-β1-receptor, Smad3, Smad7, E-cadherin, and vimentin were evaluated using quantitative reverse transcriptase–polymerase chain reaction. The protein level of E-cadherin was also determined using western blot analysis.

***Results:*** Treatment of alpha mangostin and sorafenib caused a significant decrease in the viability of sorafenib-surviving HepG2 cells versus control (both groups with *P* <0.05). Our study found that alpha mangostin treatment increased the expressions of vimentin (*P* <0.001 versus control). In contrast, alpha mangostin treatment tends to decrease the expressions of Smad7 and E-cadherin (both with *P* >0.05). In line with our findings, the expressions of TGF-β1 and Smad3 are significantly upregulated after alpha mangostin administration (both with *P* <0.05) versus control.

***Conclusion:*** Alpha mangostin reduced cell viability of sorafenib-surviving HepG2 cells; however, it also enhanced epithelial–mesenchymal transition markers by activating TGF-β/Smad pathways.

## Introduction

Hepatocellular carcinoma (HCC) is the second deadliest cancer and the fifth most common cancer worldwide. Primary treatment choices for HCC are surgical operation and hepatic transplantation. However, because of late diagnosis, most patients were unsuitable to receive those treatments. Most patients with HCC are often found at an advanced stage, which can be treated with sorafenib, the first-line approved treatment by the FDA. Sorafenib works as antiproliferation and antiangiogenesis by inhibiting several members of multikinase signaling pathways C-rapidly accelerated fibrosarcoma (C-RAF) and B-RAF and blocking proangiogenic kinases such as vascular endothelial growth factor receptors (VEGFR-2 and VEGFR-3), platelet-derived growth factors (PDGFs) receptor, c-Kit, and fims-like tyrosine kinase. However, drug resistance leads to treatment failure and, finally, the poor prognosis of the patient. At present, molecular mechanisms underlying drug resistance are poorly understood.^1-5^

One of the main causes of sorafenib resistance in liver cancer is epithelial-mesenchymal transition (EMT).^1^ The EMT is a reversible program that enables stationary epithelial cells to mesenchymal cell states. During EMT, epithelial cells that held together in the underlying basement membrane by desmosomes will lose their inter-cell connections and detach from the basement membrane. This remodeling is followed by the acquisition of mesenchymal features with increased ability to migrate, invade, and survive. Various growth factors were found to affect EMT in cancers such as hepatocyte growth factors and transforming growth factor (TGF)-β.^6-8^ TGF-β is a potent inducer of EMT. It is secreted by tumor cells and stromal fibroblasts in the tumor microenvironment.^2,8^ There is an urgent need for new active and well-tolerated treatments for advanced HCC, particularly in the case of sorafenib resistance. The addition of EMT inhibitors to sorafenib rather than sorafenib monotherapy was proposed as a solution.^9^

Alpha mangostin was known for its extensive pharmacological effect such as antibacterial, antioxidant, anti-obesity, neuroprotective agent, and anticancer.^10^ Recently, studies had reported that alpha mangostin, a xanthone isolated from *Garcinia mangostana* , exerts its antiproliferative effect by decreasing TGF-β.^11,12^

Studies using immortalized hepatic stellate cells, LX2 cells, alpha mangostin was shown to have an antifibrotic effect by alleviating TGF-β/Smad and ERK 1/2 pathways.^13,14^ Thus, alpha mangostin is predicted to have EMT inhibitory properties. Reports in pancreatic cancer and bone osteosarcoma indicate that alpha mangostin was able to suppress EMT.^15,16^ Studies on alpha mangostin in HepG2 cells have been done before by Wudtiwai et al and Mohamed et al.^17,18^ However, none have investigated the effect of alpha mangostin in EMT through TGF-β/Smad pathways in the sorafenib-surviving liver carcinoma model. Thus, the exact molecular mechanism of alpha mangostin is still poorly understood.

Therefore, this study was intended to find alpha mangostin effect on cell viability, EMT, and TGF-β/Smad signaling pathways.

## Materials and Methods

### Materials

Human HCC cells, HepG2, were gifted from the Eijkman Institute for Molecular Biology Laboratory, Indonesia. Sorafenib and alpha mangostin were purchased from St. Cruz Biotechnology. Dimethylsulfoxide was from Sigma Aldrich (Singapore). TGF-β1 ELISA kit was from R&D System (USA). Dulbecco’s Minimal Essential Medium (DMEM), fetal bovine serum - heat-inactivated, Fungizone, and Penicillin/Streptomycin were obtained from Gibco (USA). MTS assay kit was obtained from Promega (USA). Blood/Cell Total RNA mini kit was purchased from Geneaid (USA). ReverTra Ace quantitative reverse transcriptase–polymerase chain reaction (qRT-PCR) master mix with gDNA remover and Thunderbird SYBR qPCR Mix was from Toyobo (Japan). Cell lysis buffer was obtained from Invitrogen (USA). Primers used were purchased from First Base (Singapore). Primary antibodies E-cadherin, β-actin, and secondary antibody anti-rabbit IgG HRP-linked antibody were from Cell Signaling Technology (USA).

### Methods

#### Establishment of sorafenib-surviving HepG2 cells

HepG2 cells were grown until 70%–80% confluent at 37°C in a 5% CO_2_ air atmosphere in the DMEM-complete medium. Sorafenib-surviving cells were generated by exposing cells to sorafenib 10 µM for 24 hours. Subsequently, the medium was removed. All surviving cells after treatment of sorafenib were accounted as sorafenib-surviving HepG2 cells (HepG2^surv^). Control cells (HepG2^ctr^) were HepG2 naïve cells cultured in standard culture medium without sorafenib. This method was used based on the previous study using doxorubicin by Buschauer et al.^19^

#### MTS assay

HepG2^ctr^ cells and HepG2^surv^ sorafenib cells were seeded at a density of 1 × 10^4^ cells/well into 96-well plates and then treated with various concentrations sorafenib for 24 hours (0 to 20 μM). Assessment of cell viability was done using 3-(4,5-dimethyl-thiazol-2-yl)-5-(3-carboxymethoxyphenyl)-2-(4-sulfophenyl)-2H-tetrazolium MTS assay (Promega, USA) according to manufacturer’s instruction. From this method, we obtained 50% of the concentration (CC50) of both cells. Alpha mangostin dose was determined using the same techniques, with levels ranged from 0 to 30 μM.

#### Cell treatment

The treatments were divided into four groups, each group seeded with 2 × 10^6^ cells in a 10 cm culture dish. Every group was given sorafenib 10 µM for 24 hours (HepG2^surv^). Afterward, the medium was changed and then treated with vehicle only, sorafenib 10 µM, alpha mangostin 20 µM or combination of sorafenib 10 µM and alpha mangostin 20 µM for 24 hours. Subsequently, the cells were observed for their morphology and then harvested for analysis of cell viability, RNA, and protein isolation. Cell viability was assessed with trypan blue exclusion method.

#### Quantitative reverse-transcriptase PCR analysis

Blood/Cell Total RNA mini kit from Geneaid used to isolate RNA from 10^6^ cells. Subsequently, the RNA synthesized to cDNA. The primer sequence for Smad7 is mentioned as follows: Smad7 Fwd: 5′- AAACAGGGGGAACGAATTATC-3′; Smad7 Rev: 5′- ACCACGCACCAGTGTGAC-3′. On the other hand, the primer sequence for other gene were quoted from other previous studies: β-actin and Smad3 from Rahmaniah et al,^13^ TGF-β1 and TGF-β1-Receptor from Lestari et al,^14^ and E-cadherin and vimentin from Paramita et al.^20^ The qRT-PCR was done using Toyobo Thunderbird SYBR qPCR Mix (Japan) according to manufacturer protocol. Reactions were performed in qRT-PCR Light Cycler Nano Roche^TM^. Temperatures in this study are set to incubation at 95°C for 10 minutes, followed by 45 cycles of 95°C for 20 seconds and annealing temperature for 58°C–60°C for 1 minute. All quantification cycle (Cq) will be processed according to Livak method.^20^

#### Western blot analysis

The expression of E-cadherin was measured by western blot. Bio-rad protein assay used for determining protein concentration. Protein (70 μg) was separated using 7.5% sodium dodecyl sulfate (SDS)–polyacrylamide gel electrophoresis. For β-actin, the SDS page used was 10%. Afterward, the gel was transferred to nitrocellulose membranes. The membrane was then blocked with defatted milk briefly. After blocking, blots were incubated with the primary antibody for E-cadherin at 4°C overnight. Blots were washed with TBST and incubated with a secondary antibody (1:5000) for 1 hour at room temperature. Visualization of protein bands using enhanced chemiluminescence and gel scanner. The data were quantified using ImageJ software.

#### TGF-β1 concentrations in culture medium

The TGF-β1 medium concentration was examined with the Human TGF-β1 ELISA kit (R&D System) following kit protocol.

#### Statistical analysis

Results were presented as mean ± SEM. Analysis of all data distribution and variants done before statistical analysis. Normally distributed data were analyzed using one-way ANOVA, followed by post hoc analysis using the Tukey method or one-way Welch’s ANOVA—post hoc Games–Howell analysis. Data with non-normal distribution were analyzed using Kruskal–Wallis followed by post hoc Tukey or Mann–Whitney method. Statistically significant difference was set at *P* < 0.05. GraphPad Prism version 8 (USA) used to produce all the graphs in this study.

## Results and Discussion

### CC50 of sorafenib-surviving HepG2 cells (HepG2^surv^) 

**Figure 1:**
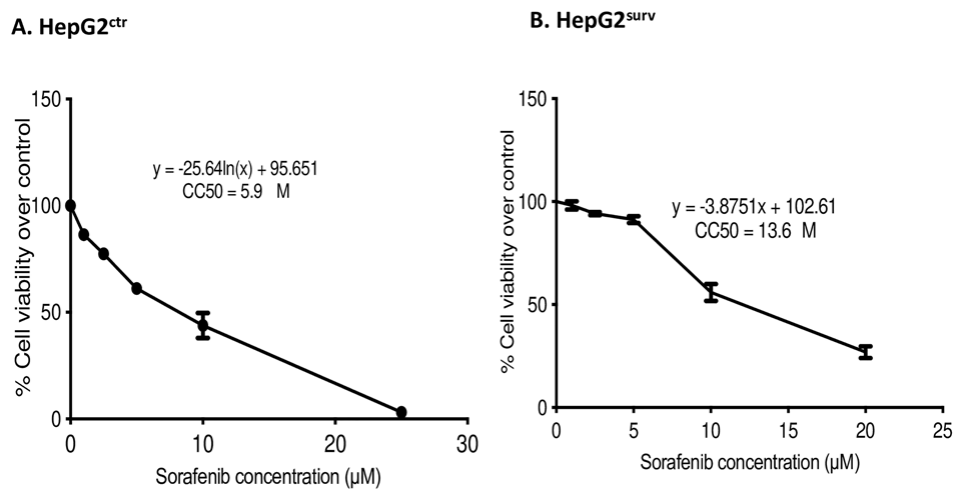
CC50 of (A) HepG2^ctr^ and (B) HepG2^surv^ after 24-hour treatment with sorafenib at 0, 1, 2.5, 5, 10, and 20 µM.

This study aims to investigate alpha mangostin role on human HCC cells surviving sorafenib in vitro, in terms of TGF-β/Smad-related epithelial–mesenchymal activation. To achieve our aim, we established HepG2^surv^ sorafenib cells. We compared 50% cytotoxicity concentration (CC50) of HepG2^ctr^ and HepG2^surv^ cells (Figure 1). Sorafenib-surviving cells (HepG2^surv^) require 2.3-fold in sorafenib concentration to give the same effect than naïve HepG2 cells (HepG2^ctr^).

After the treatment of sorafenib, the surviving cells become less susceptible to retreatment with sorafenib 10 µM as shown by the increase in CC50 HepG2^surv^ cells as compared with HepG2^ctr^. However, sorafenib 10 µM is the highest achievable clinical concentration.^21^ Thus, to achieve a higher effect, the dose of sorafenib could not be further increased. The previous study has proved the antiproliferative potential of alpha mangostin by G1/S cycle arrest, apoptosis mediated by caspase-independent pathways, and topoisomerase inhibition.^12,18,22^ Alpha mangostin also can reduce Akt and ERK 1/2 pathways.^14^ All-of-the-above mechanisms explained that alpha mangostin administration, whether single or in combination with sorafenib, can reduce HepG2^surv^ cell viability.

### Determination of alpha mangostin dose in HepG2surv

**Figure 2:**
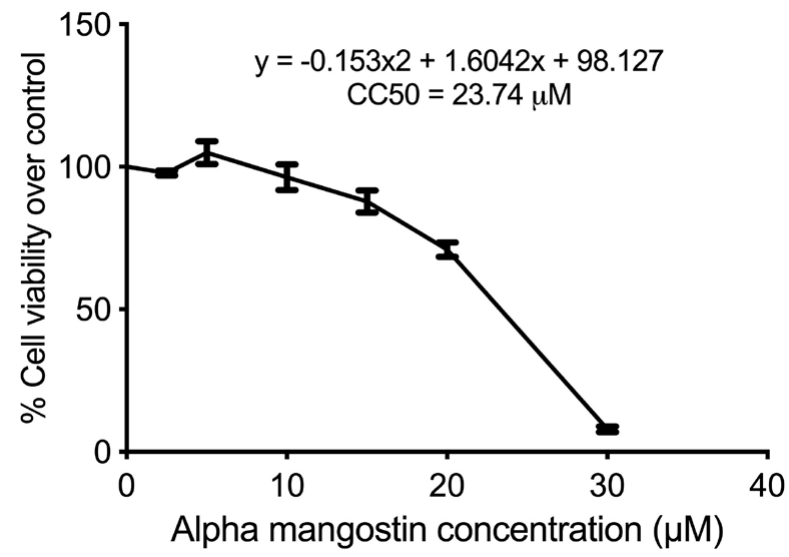
CC50 of sorafenib surviving HepG2 cells (HepG2^surv^) cells after 24-hour treatment with alpha mangostin at 0, 2.5, 5, 10, 15, 20, and 30 µM.

Alpha mangostin dose used in the experiment was the dose that resulted in the reduction of about half of cell viability in sorafenib-surviving HepG2 cells (HepG2^surv^) over control. According to the CC50, as determined by the MTS assay, CC50 of alpha mangostin was 23.74 µM (Figure 2). Hereafter, we used 20 μM of alpha mangostin for further application.

### Cell viability

**Figure 3:**
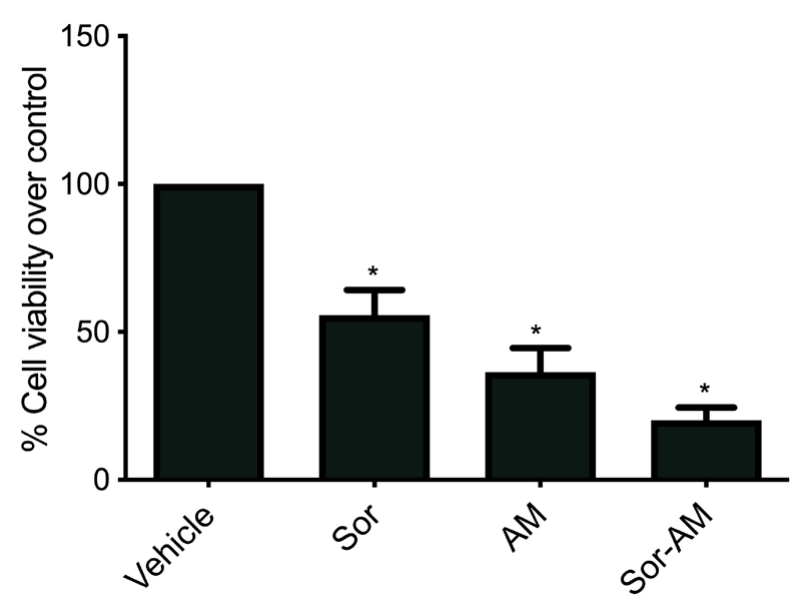
Effect of sorafenib, alpha mangostin, or combination of sorafenib and alpha mangostin in HepG2^surv^ on cell viability over control. Results were presented as mean ± SEM. *: p<0.05 versus vehicle. Vehicle: DMSO, Sor: Sorafenib 10 μM; AM: alpha mangostin 20 µM, Sor-AM: Sorafenib 10 μM + alpha mangostin 20 μM.

Sorafenib was given to sorafenib-surviving cells (HepG2^surv^), still able to decrease cell viability versus control; however, the effect was not as strong as the first sorafenib. Treatment of alpha mangostin or combination of sorafenib-alpha mangostin to HepG2^surv^ resulted in a better reduction in a decrease of cell viability (Figure 3).

The effect of alpha mangostin as an anticancer has been established in various types of human cancer. The previous study has proved that this effect happens by induction of caspase-independent and caspase-dependent apoptosis or by induction cell-cycle arrest at G1-phase and was known to be correlated with TGF-β signaling through the extrinsic apoptotic pathways. The extrinsic pathways are mediated by the interactions of ligand and death receptors, such as FasL/FasR and TNF-α/TNFR1. When Fas Ligand (FasL) is binding to the Fas receptor, then the receptor will cluster and induce the formation of death-inducing signaling complex to recruit and activate caspase 8 via adaptor molecule Fas-associated death domain protein. Caspase activity will provoke chromatin and cytoplasm condensation, fragmentation of DNA and the nucleus, cell shrinking, and finally, degradation of the cell into apoptotic bodies. Daxx, a Fas receptor-associated protein, which mediates the activation of JNK and the apoptotic cell death induced by Fas, physically interacts with TGF-β-RII and is involved in mediating TGF-β-induced apoptosis. TGF-β1 will induce the formation of Fas receptor superclusters leading to receptor activation. This is in line with a study from Lee et al, who showed alpha mangostin as a prooxidant that activated the TGF-β signaling pathways, and Kim et al, who reported that TGF-β1 may induce apoptosis in human SNU-620 carcinoma gastric cells in a Fas ligand-independent manner through Fas death pathways.^23,24^

### HepG2 cell morphology

**Figure 4:**
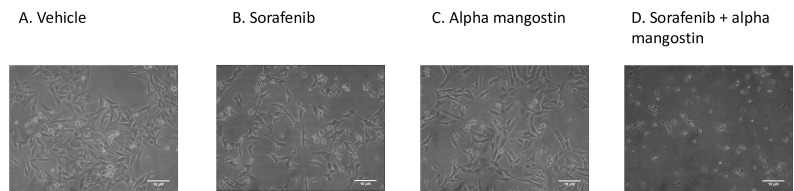
Cell morphology of sorafenib-surviving HepG2 cells (HepG2^surv^) after treatment with (A) vehicle only, or (B) sorafenib 10 µM, or (C) alpha mangostin 20 µM, or (D) sorafenib 10 µM and alpha mangostin 20 µM.Cells were photographed under a contrast phase inverted microscope at x100 magnification.

Cell morphology of sorafenib-surviving cells (HepG2^surv^) after treatment with vehicle or sorafenib or alpha mangostin or sorafenib and alpha mangostin were observed using an inverted microscope. No significant changes in morphology were found of the cells between treatment groups (Figure 4).

In our study, we did not find any difference in the morphology of HepG2^surv^ sorafenib cells after treatment with sorafenib, alpha mangostin alone, or in combination with sorafenib. All cells have lost the epithelial morphology cobblestone appearance and developed to spindle-like, outstretched mesenchymal-like shape. These indicate that EMT has likely occurred. The EMT is a phenomenon that refers to the transition of differentiated epithelial cells to mesenchymal cells in specific physiological and pathological conditions.^20^

### The effect of alpha mangostin alone or in combination with sorafenib on the expression of EMT markers, vimentin, and E-cadherin

**Figure 5:**
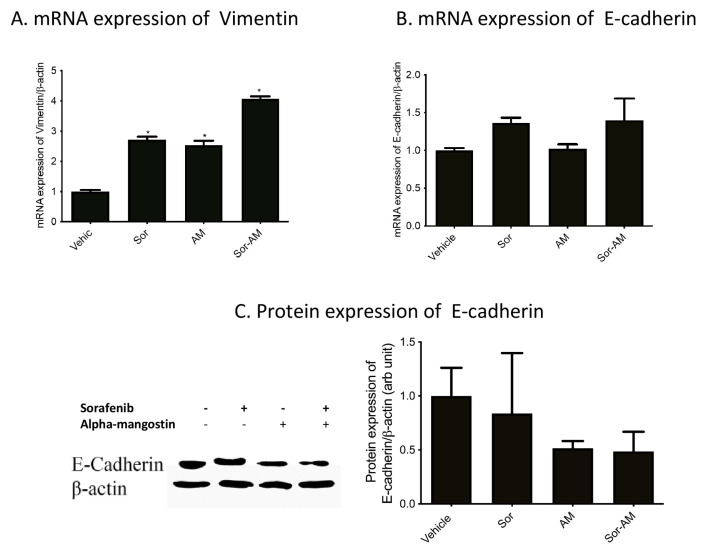
Effect of HepG2^surv^ treatment with sorafenib, alpha mangostin or sorafenib-alpha mangostin on (A) mRNA expression of vimentin/β-actin, (B) mRNA expression of E-cadherin/β-actin, (C) protein expression of E-cadherin/b-actin. Results were presented as mean ± SEM. *P < 0.05 versus vehicle, #P < 0.05 versus sorafenib. Vehicle: DMSO; Sor : Sorafenib 10 μM ; AM: alpha mangostin 20 μM, Sor-AM : Sorafenib 10 μM + Sorafenib 10 μM – alfa mangostin 20 μM.

Sorafenib and alpha mangostin given to HepG2^surv^ escalate mesenchymal marker expression markers that are shown by vimentin mRNA expression. However, no statistical significance between treatment was found for mRNA expression and protein expression of E-cadherin.Yet we observed a tendency toward the reduction of both E-cadherin mRNA and protein in HepG2^surv^ treated with the combination of sorafenib and alpha mangostin (Figure 5).

In addition to cell morphology, the EMT process can also be shown with markers. Hallmarks of EMT include the loss of E-cadherin expressions, in conjunction with the increase in vimentin expression.^25^ Vimentin is an intermediate filament protein expressed by mesenchymal cells. This protein has been used widely as a mesenchymal marker. Interestingly, in this study, alpha mangostin alone, or in combination with sorafenib, increases the mRNA expression of vimentin.

E-cadherin is an adherens junction protein that plays a role in the establishment and maintenance of cell polarity, differentiation, migration, and signaling in cell proliferation pathways. E-cadherin forms intercellular contacts in epithelial cells and interacting with intracellular cytoskeletal networks.^26^ The loss of E-cadherin contributes to the increased cell invasion and metastasis in carcinoma. In our study, we found a slight decrease in E-cadherin expression after treatment with alpha mangostin. We assumed that partial EMT occurred in our cells, as sorafenib also suppressed EMT. As previously known, among the three main different types of EMT (EMT on embryogenesis, EMT on chronic fibrotic pathologies, and EMT during tumor progression), the tumor-related EMT most often undergoes an incomplete or partial EMT.^27-30^

We found different results from previous studies conducted by Xu et al in osteosarcoma MG-63 cells and Park et al in pancreatic cancer cells. Both studies showed that alpha mangostin suppressed EMT.Xu et al reported that alpha mangostin treatment in BxPc-3 and Panc-1 cells resulted in the reduction of EMT markers by inhibiting PI3K/Akt pathways,^15^ whereas Park et al showed that alpha mangostin inhibit MAPK Pathways (ERK 1/2, JNK, and p38).^16^ However, Park et al also showed that although E-cadherin expressions were increased, Snail protein expression in the dose of 10 µM was elevated significantly.^16^ We assume that the differences in the findings from this study with Xu et al and Park et al were most likely due to the difference in cell types.^15,16^ The fact that their research was done in naïve cells were the main thing that contributes to this distinction. The difference between naïve cells and resistant cells was explained by Chen et al, who found a different downstream of the MAPK signaling pathway in untreated versus sorafenib-treated HCC cell lines. Sorafenib-resistant HCC cells induced upregulation in c-Jun expression as compared with sorafenib-sensitive cells.^31^ Aisha et al also reported that alpha mangostin exerted different effects on different tumor cells.^32^ In line with our result, Aisha et al reported that in human colorectal carcinoma, xanthone extracts from *Garcinia mangostana* upregulated the expressions of MAPK/ERK, MAPK/JNK, c-Myc/Max, and p53 after alpha mangostin treatment.^14-16,26,31,32^

### The result of alpha mangostin alone or in combination with sorafenib on the expression of mRNA TGF-β1, mRNA TGF-β-receptor I, and concentrations of TGF-β in culture medium

**Figure 6:**
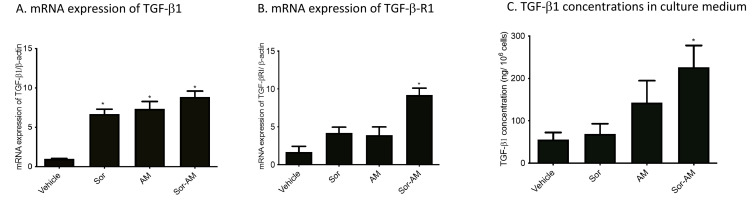
Effect of HepG2^surv^ treatment with sorafenib, alpha mangostin, or combination of sorafenib-alpha mangostin on (A) mRNA expressions of TGF-β1/β-actin, (B) mRNA expression of TGFβ-receptor 1/β-actin, and (C) TGF-b1 concentrations in a culture medium. Results were presented as mean ± SEM. * *P*  < 0.05 versus vehicle, #*P*  < 0.05 versus sorafenib. Vehicle: DMSO; Sor: Sorafenib 10 μM; AM: Alpha mangostin 20 μM; Sor-AM: Sorafenib 10 μM + alfa mangostin 20 μM.

All of the treatment has been shown to increase mRNA expressions of TGF-β1 significantly versus control (vehicle). However, the increase of mRNA expressions of TGF-β-Receptor 1 and concentrations of TGF-β1 in culture medium was only apparent in HepG2^surv^ treated with combinations of sorafenib and alpha mangostin (Figure 6).

EMT process requires the activation of cytokines, such as PDGF, fibroblast growth factor, TGF-β and tumor necrosis factor-α (TNF-α). Among the cytokines, TGF-β is the most potent EMT-inducing agent.^33^ TGF-β orchestrates a variety of cellular processes such as cell differentiation, proliferation, cycle arrest, survival, and adhesion, as well as extracellular matrix production. In our study, we detected the consistent increase in TGF-β concentrations in the medium, along with the upregulation of TGF-β1 mRNA expression and the TGF-β1 receptor after sorafenib-surviving cells were treated with alpha mangostin and sorafenib. Our result is in line with Kang et al, who reported that TGF-β expressions increase in HCC sorafenib resistant cell line.^34^ We think that the upregulation of mRNA expressions TGF-β takes place because there is multiple signaling regulated EMT. The two major pathways in EMT are Smad-dependent pathways and non-Smad dependent pathways, which communicate and regulate each other. Their crosstalk signaling mechanism until today still not fully understood. The blocking in one receptor signaling pathways will be compensated by rewiring to other pathways. Several studies focused on inhibiting specific RTK have proved despite showing a favorable initial response; they then led to an unsatisfactory result.^35^ Other studies also showed initial or acquired resistance was caused by cross-talk between VEGFR/MAPK and PI3K/Akt or JAK-STAT.^2,36,37^

Our data also showed that mRNA TGF-βRI expression increase after sorafenib and alpha mangostin administration versus control. Although previous studies show, sorafenib can inhibit the TGF-β-induced EMT induced by downregulating cell-surface type II TGF-β receptors (TGF-β-RII); long-term exposure to sorafenib leads to an epithelial-to-mesenchymal transition.^38^ These are likely due to molecular pathways changes in HepG2-resistant sorafenib cells. As known, sorafenib is a multi-kinase inhibitor whose targets mainly include VEGFR, rapidly accelerated fibrosarcoma kinase, PDGF receptor, and c-Kit. By suppressing these pathways, another compensatory survival mechanism has been activated. Lestari et al reported an alpha mangostin effect in hepatic stellate cells activation by decreasing expression of TGF-βR mRNA. Alpha mangostin also is known as suppressing the viability and EMT by downregulating the PI3K/Akt pathway in pancreatic cancer cells.^14,15^

In contrast to our result that showed alpha mangostin increased TGF-β pathways in sorafenib-surviving cells, Rahmaniah et al and Lestari et al reported an inhibitory effect of alpha mangostin on TGF-β/ERK and TGF-β/SMAD pathways. However, their research was done in hepatic stellate cells, which is a non-tumor cell and did not investigate its effect on EMT.^13,14^

The expression of mRNA TGF-βRI after the combination of sorafenib and alpha mangostin compared with sorafenib treatment also significantly increases. These results are probably due to the alpha mangostin effect on TGF-β noncanonical signaling. Akao et al have demonstrated that alpha mangostin can activate ERK 1/2 signaling in colon cancer cells. This result suggests that in sorafenib-resistant cells, alpha mangostin and sorafenib work synergistically in TGF-β noncanonical pathway by upregulating TGF-βR.^39^ Previous reports have implicated that MAPK pathways could mediate TGF-β-induced EMT.^39,40^

### The effect of alpha mangostin alone or in combination with sorafenib on the mRNA expression of Smad3 and Smad7

**Figure 7:**
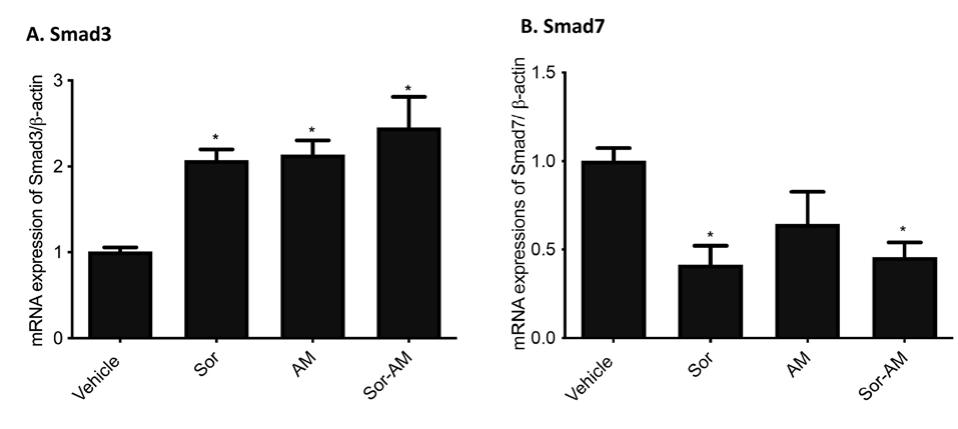
Effect of HepG2^surv^ treatment with sorafenib, alpha mangostin, or sorafenib-alpha mangostin on mRNA expressions of (A) SMAD3/β-actin and (B) SMAD7/β-actin. Results were presented as mean ± SEM. *P < 0.05 versus vehicle. Vehicle: DMSO; Sor: Sorafenib 10 μM; AM: alpha mangostin 20 μM, Sor-AM: Sorafenib 10μM + Sorafenib 10 μM – alfa mangostin 20 μM.

We found a significant increase in SMAD3 after HepG2^surv^ were treated with alpha mangostin, both alone and in combination with sorafenib administration. In contrast, the expression of mRNA SMAD7 tends to decrease after treatment with sorafenib, alpha mangostin, or sorafenib–alpha mangostin combination (Figure 7).

The results show that TGF-*β* 1 activates the Smad pathway. Upon stimulation from an active form of TGF-β1, TGF-β1 receptor types I and II (TGF-β-RI and TGF-β-RII) will form heteromeric complexes and phosphorylated R-Smads (Smad2 and Smad3). The Smad2 and Smad3 then assemble with Smad4 into a complex and translocates to the nucleus. In the nucleus, they bind to DNA and interact with transcription factors activating or repressing target genes.^41^ This study showed that alpha mangostin and sorafenib increase the expression of TGF-β/Smad pathways, as indicated by enhancement Smad3 and vimentin expression.

Moreover, Smad7, which prevents Smad2 and Smad3 interaction by forming a stable complex with activated TGF-β-RI, also decreases after sorafenib and alpha mangostin administration.^42^ In this study, the proteomic analysis of Smad3 and Smad7 were not available. However, previous studies have proved that the mRNA and protein expressions of Smad3 and Smad7 were parallel, so it would not change the interpretation.^43,44^

## Conclusion

In conclusion, alpha mangostin alone and in combination with sorafenib have the ability to suppress cell viability of sorafenib-surviving HepG2 cells. However, alpha mangostin tends to increase EMT through TGF-β/Smad pathways. Thus, specific mechanism of alpha mangostin in attenuating sorafenib resistance in HCC still needs to be explored.

## Ethical Issues

This study used HepG2 cell line that was gifted by the Eijkman Institute for Molecular Biology. This work did not involve direct access to any human samples and was not subject to human ethics review.

## Conflict of Interest

Authors declare no conflict of interest in this study.

## Acknowledgments

This study was funded by QQ Grant from the Directory of Research and Community Engagement, Faculty of Medicine, Universitas Indonesia. The authors would like to thank Enago (https://www.enago.com) for the English language review.
